# Lombards on the Move – An Integrative Study of the Migration Period Cemetery at Szólád, Hungary

**DOI:** 10.1371/journal.pone.0110793

**Published:** 2014-11-04

**Authors:** Kurt W. Alt, Corina Knipper, Daniel Peters, Wolfgang Müller, Anne-France Maurer, Isabelle Kollig, Nicole Nicklisch, Christiane Müller, Sarah Karimnia, Guido Brandt, Christina Roth, Martin Rosner, Balász Mende, Bernd R. Schöne, Tivadar Vida, Uta von Freeden

**Affiliations:** 1 Center for Natural and Cultural History of the Teeth, Danube Private University, Krems, Austria; 2 State Office for Heritage Management and Archaeology Saxony-Anhalt and State Museum of Prehistory, Halle, Germany; 3 Institute for Prehistory and Archaeological Science, Basel University, Basel, Switzerland; 4 Curt Engelhorn Centre Archaeometry gGmbH, Mannheim, Germany; 5 Institut für Prähistorische Archäologie, Freie Universität Berlin, Berlin, Germany; 6 Department of Earth Sciences, Royal Holloway University of London, London, United Kingdom; 7 Laboratório Hercules, Universidade de Évora, Évora, Portugal; 8 Institute of Anthropology, University of Mainz, Mainz, Germany; 9 IsoAnalysis UG, Berlin, Germany; 10 Archaeological Institute, Research Centre for Humanities, Hungarian Academy of Sciences, Budapest, Hungary; 11 Institute of Geosciences, University of Mainz, Mainz, Germany; 12 Department of Prehistory and Protohistory, Eötvös Loránd University of Budapest, Budapest, Hungary; 13 German Archaeological Institute, Roman Germanic Commission, Frankfurt a. M., Germany; Museo Nazionale Preistorico Etnografico 'L. Pigorini', Italy

## Abstract

In 2005 to 2007 45 skeletons of adults and subadults were excavated at the Lombard period cemetery at Szólád (6^th^ century A.D.), Hungary. Embedded into the well-recorded historical context, the article presents the results obtained by an integrative investigation including anthropological, molecular genetic and isotopic (δ^15^N, δ^13^C, ^87^Sr/^86^Sr) analyses. Skeletal stress markers as well as traces of interpersonal violence were found to occur frequently. The mitochondrial DNA profiles revealed a heterogeneous spectrum of lineages that belong to the haplogroups H, U, J, HV, T2, I, and K, which are common in present-day Europe and in the Near East, while N1a and N1b are today quite rare. Evidence of possible direct maternal kinship was identified in only three pairs of individuals. According to enamel strontium isotope ratios, at least 31% of the individuals died at a location other than their birthplace and/or had moved during childhood. Based on the peculiar ^87^Sr/^86^Sr ratio distribution between females, males, and subadults in comparison to local vegetation and soil samples, we propose a three-phase model of group movement. An initial patrilocal group with narrower male but wider female Sr isotope distribution settled at Szólád, whilst the majority of subadults represented in the cemetery yielded a distinct Sr isotope signature. Owing to the virtual absence of Szólád-born adults in the cemetery, we may conclude that the settlement was abandoned after approx. one generation. Population heterogeneity is furthermore supported by the carbon and nitrogen isotope data. They indicate that a group of high-ranking men had access to larger shares of animal-derived food whilst a few individuals consumed remarkable amounts of millet. The inferred dynamics of the burial community are in agreement with hypotheses of a highly mobile lifestyle during the Migration Period and a short-term occupation of Pannonia by Lombard settlers as conveyed by written sources.

## Introduction

Historical references to the Lombards or Longobards date back to the first century A.D. during which this *gens* inhabited the lower Elbe region in northern Germany [1]–[3]. In the second century they disappear from the Roman sources until the late 5^th^ century, when reference to “Longobards” reoccurs in the region north of the middle Danube. There is fierce debate as to whether these “new” Longobards had anything in common with and whether there was any continuity between them and those mentioned during the Roman period, or whether a second ethnogenesis took place and resulted in a group that adopted the old name. Subsequent Roman and Byzantine sources as well as later records produced by Romanised Christian authors report on the Lombards being involved in battles, and in events of resettlement and land seizures, initially on the middle Danube in Moravia and lower Austria, and subsequently in Pannonia. Finally, and – most notably – the records describe their final invasion into Italy [4]–[6]. The history of the Lombards has been appraised as a key example of a protohistoric migration. It took place at the very end of the Great Germanic Migrations across Europe and involved only three generations approximately. It started around A.D. 500 with the reoccurrence of their name north of the middle Danube outside the former Roman Empire and ended in A.D. 568 with the foundation of the Lombard kingdom in Italy which marked the end of the period of the Great Migrations.

Pannonia was the core area of Lombard settlement in the 6^th^ century. Historical sources provide accounts of the *gens* occupying the north of the former Roman province of Pannonia in A.D. 526 and the south in A.D. 546; they also mention the year of their migration to Italy [1]. Archaeologically, some 40 cemeteries containing approximately 2,000 burials are known from the Lombard period [7]. They have yielded evidence of funerary customs and characteristics of material culture that are comparable to those in the surrounding areas and the regions around the postulated route of migration. This has raised questions and hypotheses regarding the identification and social structure of the Lombards as well as the impact of residential relocation. Despite snippets of information from written records and archaeological evidence it still remains unclear whether any migrations of larger groups took place, or whether the sources were biased by military interests and thus possibly exaggerated the role played by mass movement. It is furthermore unknown whether incoming groups had already been affiliated for several generations, or whether they were only loosely connected and incorporated members of different backgrounds and ancestry, as is known to have been the case with “the” Lombards when they arrived in Italy. Likewise, the relationships between the local populations and the new arrivals in Moravia, Pannonia and later in Italy remain unknown [8].

The cemetery at Szólád, Komitat Somogy, which dates from the 6^th^ century A.D., is a location with excellent conditions for an in-depth investigation into the Lombard occupation of Pannonia. Both the rich archaeological record and the excellently preserved skeletal remains allow for a comprehensive characterisation of the burial community using an unprecedented interdisciplinary approach, which includes archaeological, anthropological, palaeogenetic and isotopic (δ^15^N, δ^13^C, ^87^Sr/^86^Sr) analyses. Strontium (Sr) isotope analysis provides information with regard to residential changes [9], [10], while carbon (C) and nitrogen (N) isotope data reflect dietary habits [11].

Recent historical and archaeological research has taken into account the heterogeneity of early medieval *gentes* such as the Lombards and any other contemporary group that carries an ethnic label. The aim of this interdisciplinary project is therefore not to characterise “the Lombards” by using archaeometric methods, but to address the following specific questions: Does this burial community represent a group of people with the same biological background? Are there any indications of first-generation immigrants, or that a group or perhaps several groups of closely related family members moved together? Did non-local people originate from the same area, or do genetic heterogeneity and variable strontium isotope ratios point to flexible group affiliations and the incorporation of people of different foreign origin? Did the migration result in stress markers that can be identified in the human skeletons, for instance nutritional stress, detrimental health conditions, or even increased occurrence of interpersonal violence? The Szólád cemetery provides excellent opportunities for a sound characterisation of the living conditions and the movement of people during the Migration Period (6^th^ century A.D.).

## The Early Medieval Cemetery at Szólád, Hungary

**Figure 1:**
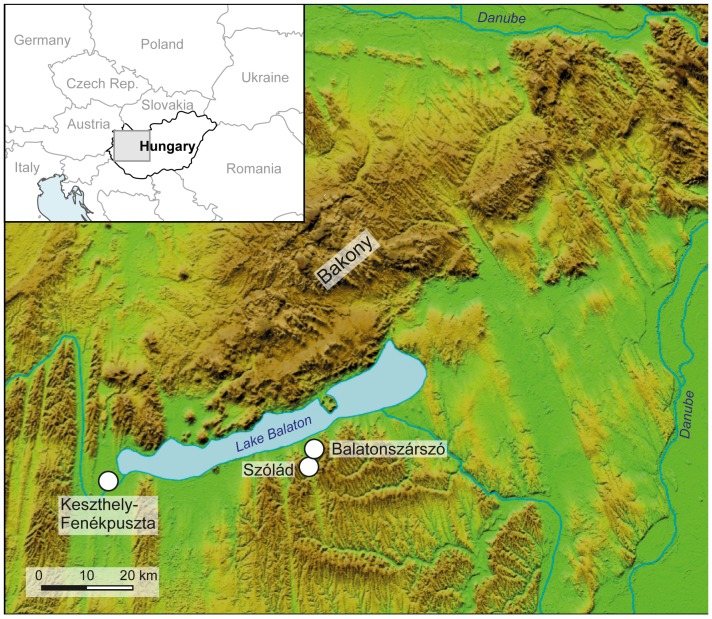
Map showing the location of the Szólád cemetery on the southern shore of Lake Balaton and the sites of Balatonszárszó and Kestzthely-Fenékpuszta, which have yielded strontium isotope reference data. The DEM is based on SRTM (90 m) data, edited by H.-J. Köhler, U. v. Freeden, D. Peters and C. Knipper.

Szólád is situated approximately five kilometres south of Lake Balaton on a south-oriented loess slope [12] above a valley that is 30 km long, and at Szólád some 400 to 600 m wide. The area is a boggy former backwater of the lake [13] (Fig. 1). Excavations took place between 2005 and 2007 as a joint venture of the Roman-Germanic Commission (RGK) of the German Archaeological Institute in Frankfurt am Main (Germany), the Committee of Archaeology of the Hungarian Academy of Sciences in Budapest (Hungary), and the Institute of Anthropology of the University of Mainz (Germany). Planning, fieldwork, and analysis were conducted in close collaboration and the early involvement of anthropologists ensured an appropriate recovery of the human skeletal remains and sterile *in situ* sampling.

**Figure 2:**
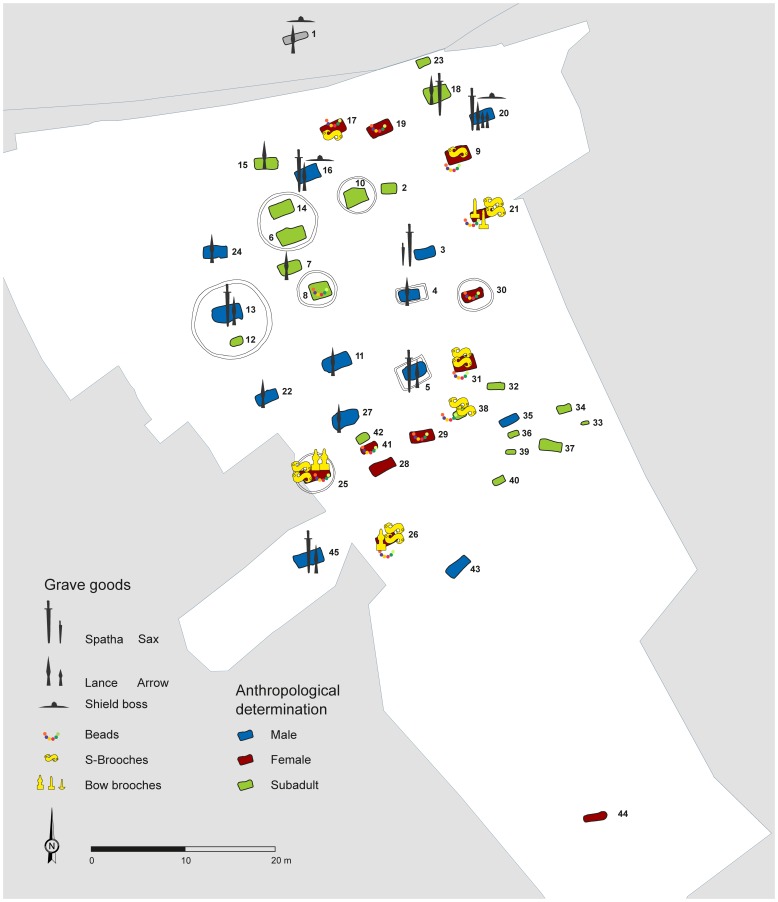
Plan of the cemetery at Szólád with the locations of selected grave goods. The colours of the graves symbolise the anthropological sex and age determinations (graphics: U. v. Freeden, D. Peters, C. Knipper).

The cemetery yielded 45 inhumations from the Lombard period (Fig. 2). Based on stylistic elements the grave goods were dated to between the second third and second half of the 6^th^ century A.D., which is in accordance with the occupation of the southern part of Pannonia by the Lombards recounted by historical records [5], [7], [14]. There was remarkable variation with regard to the construction of the graves: Eleven (numbers 19, 28, 32, 33, 35–40, 43) had straight vertical walls, whilst the remaining 34 burials were so-called “ledge graves” with ledges in the side walls that supported wooden beams covering the coffins, which were either chest-like or actual tree-trunk coffins. The pits of women's and children's graves tended to be shallower than those of the men's burials, which were dug up to more than four metres deep into the loess. Six circular ditches surrounded one or two graves each (Ind. 8, 10, 25, 30, 6/14, 12/13). Such features are known from other Lombard period cemeteries at Holubice, Holásky, Smolín, and Lužice in Moravia [15]–[17]. Rectangular enclosures surrounding graves 4 and 5 are also worth mentioning and suggest that there was some sort of a relationship between the deceased. While only a few parallels are known in the West [18], such features may point to Roman traditions [19]; given that the site was situated within the former Roman Empire, this would be an element specific to Szólád. In addition to the elaborate grave constructions, the burials stood out by virtue of their lavish grave goods, including high-quality brooches and weaponry (Text S1). They had survived extensive disturbances typical of cemeteries from the Lombard period [13]; [20]. The presence of grave goods in nearly all the burials not only allowed archaeologists to date the finds precisely, but also reflected a need for self-representation and perhaps suggests that social differentiation existed among the members of the burial community. The males' graves were concentrated in the western area of the site, whilst the females' graves were arranged in a half circle around them on the eastern side. Children were buried in two groups in the northern and south-eastern parts of the cemetery. Some of the children's graves in the south-east did not contain any objects. The same applied to the inhumations of the juvenile individual 37 and the adult male individual 43 in the southern periphery of the cemetery, as well as the young adult male individual 44, whose ledge grave was situated at a distance of 20 metres from the burial site. Because of the lack of objects this intentional separation is difficult to explain and may have been due to social reasons, although a chronological separation, e. g. inhumation in the subsequent Avar period, cannot be excluded either. Apart from this, there was no indication of any chronological differentiation within the cemetery: All graves contained mid-6^th^ century material. Since the historical sources suggest that the Lombards only stayed in southern Pannonia for approximately twenty years, all adult individuals buried at Szólád would have been non-local to the site. This hypothesis lay at the core of the study of the cemetery.

## Sampled Materials

The study included skeletal remains of 44 out of a total of 45 interments which represent the burial community from the Lombard period (Fig. 2, File S1). The calcareous loess ensured favourable conditions for skeletal preservation both at a macroscopic and a biomolecular level (cf. Figures a and b in Text S1). The investigation of teeth and bones provided essential anthropological information such as age, sex, body height, traces of injuries and diseases as well as stress markers [21]. These data clarified the demographic structure and epidemiological conditions and were the basis for the interpretation of the results obtained from the isotopic and molecular genetic investigations (Table A – J in File S1).

Immediately after the skulls were uncovered by the excavators, four or more teeth – preferably molars and incisors – were extracted from each individual *in situ* for biomolecular analyses [22]. In a few cases additional sampling in the laboratory was required. Owing to incomplete tooth development or bad preservation, tooth samples from some of the very young children in particular could not be processed for aDNA analysis. This reduced the sample for the molecular biological study to 38 individuals of which at least two different teeth were analysed in each case (Table K and L in File S1).

Wherever possible, strontium isotope analyses were carried out on pairs of teeth from the permanent dentition of the same individual, namely the enamel of a second incisor, canine or first molar, which form in the first four years of life, and a third molar, which represents late childhood and adolescence [23]. The data regarding the children that had died before permanent dentition had formed were retrieved from deciduous teeth, and second molars or premolars were sampled in adults and older children that did not have any third molars. Depending on the type of tooth, the bulk samples averaged an isotope signal from about 1.5 to just over 3 years. Extracting the sample material for strontium separation from a reservoir of enamel that originated from the whole preserved crown height reduced the noise in the data compared to sampling random portions of the tooth crowns.

In total, the Sr isotope dataset (Table M in File S1) comprises 66 enamel samples from 35 individuals. Two human bones from the cemetery (Ind. 4 and 17), 21 samples of modern ground vegetation and four water samples collected from the main geological units in the vicinity of Szólád (up to a distance of 12 km) and in the wider areas north and south of Lake Balaton provided comparative data for the interpretation of the ^87^Sr/^86^Sr ratios of the human teeth (Table N and Figure A in File S1). Additionally, ten soil samples from fields located at a distance of approximately 100 m around the cemetery and from gardens up to 450 m away were also analysed. They potentially represent the fields farmed by the Lombard community, but may be contaminated by modern agricultural soil management.

Collagen for carbon and nitrogen isotope analysis was preferably extracted from rib fragments or, if no suitable rib bones were available, from long bones (Table M in File S1). Bones of domestic pigs, sheep/goat, cattle, chickens and geese – all found in the Lombard graves – served as comparative samples (Table O in File S1).

## Methods

The assessment of the complex processes that occurred in each archaeological context requires an interdisciplinary approach which must include anthropological and archaeometric investigations. The osteological study, ancient DNA analysis, sample preparation and measurement of stable carbon and nitrogen isotope compositions of the bone collagen, as well as the strontium isotope ratios of tooth enamel, water and modern vegetation followed established methods with minor modifications, which are all described in the supporting material (Text S2). The online material also includes lists of all specimens analysed (File S1). The human and faunal skeletal remains from Szólád, Komitat Somogy, Hungary are in storage at the Hungarian Academy of Sciences, Research Centre for the Humanities, Archaeological Institute, Budapest. The excavation permits were issued by the Office of Cultural Heritage in Budapest and are kept in the archives of the authority.

## Results and Discussion

### Osteology

Table B in File S1 illustrates the demographic composition of the burial community of Szólád: 19 individuals (43.2%) died before reaching adulthood, whilst 25 individuals were adults at the time of their death (56.8%). Contemporary cemeteries in south-eastern Europe yielded either similarly high proportions of subadult individuals (Hungary: Hegykő [39,5%], Rácálmas-Újtelep [47%]; Slovakia: Bratislava-Rusovce [45%]; [24], [25]) or considerably lower values averaging around 15% (Hungary: Tamási, Szentendre, Kajdacs-Homokbánya [24]) (Table C in File S1). Cemeteries of a similar size dating from the 6^th^ century A.D. in south-western Germany and Switzerland exhibit lower infant mortality rates, which could, however, also have been due to the burial practices, which may have resulted in a deficit of children of the age group infans I (e. g. Hemmingen [17%], Heidenheim [20%], Basel-Bernerring [15.6%], Giengen a.d. Brenz [6.8%] [26]). The sex ratio of the adults at Szólád was quite balanced. On average, females died at a younger age than males, similar to observations made among earlier prehistoric and historical populations [27].

The average body height was 156.5±3.5 cm for females (n = 9) and 169.9±3.5 cm for males (n = 11). Two females fell into the overlapping range between males and females; all other females were shorter than the males (Table A in File S1). Other Lombard and Thuringian populations from Hungary [28] and Germany [29] yielded similar average values within a range of between ca. 2 cm (males) and 4 cm (females) (Table D in File S1).

Four skull fractures and eight traumata on the postcranial skeleton were identified in a total of eight adults and one juvenile individual (Table E in File S1). The skull injuries were exclusive to male remains and included three cases of sharp-force trauma (Ind. 4, 13, 27) as well as one case of a depressed fracture (Ind. 43). Three skull fractures bore traces of healing, whilst one had occurred around the time of death (Ind. 13) (Text S3). Two females (Ind. 30 and 31) and four males (Ind. 11, 27, 37, 45) bore a total of seven healed and one unhealed fractures (Ind. 37) on the postcranial skeleton: two humeri (Ind. 11, 45), a femur (Ind. 31), a tibia (Ind. 37), a rib (Ind. 11), a toe (Ind. 30) as well as an ulna and a patella (Ind. 27). Only the femur fracture had caused a marked deformity in the leg (Ind. 31). The frequency of fractures (nine fractures on 44 individuals, i.e. 20.5%) identified in the burials studied at Szólád was above the usual average observed in early medieval populations [30].

Degenerative diseases, which were quite common in prehistoric times [31], included moderate cases of age-related osteoarthritis or spondylosis (Table A and Table F in File S1). The bones of eight males and six females of 22 observable adult individuals were affected by osteoarthritis. With seven females and ten males affected, the frequency of spondylosis was somewhat higher, and more individuals suffered from quite severe forms of the disease. Schmorl's nodes as further evidence of physical activity occurred on the vertebrae of three females and six males. Degenerative wear was diagnosed in almost all adults of the Szólád burial community, and its severity was directly proportional to the individual's age at death. In some cases it coincided with ankylosis (Text S3).

Periosteal lesions and Cribra orbitalia are nonspecific stress markers usually linked to malnutrition and infectious diseases [32], [33]. Infectious reactions were identified on the skulls of two males and seven subadult individuals. The skeletons of the subadults revealed periosteal lesions on the cranial vaults, orbitae, palates, and mandibles (Table A and Table G in File S1). Periosteal lesions on the postcranial skeleton occurred in four adults and four subadults, which represents approximately 50% of the children and 20% of the adults (Table H in File S1). Cribra orbitalia was observed in a total of 16 out of 44 individuals (Table A and Table I in File S1). This frequency is similar to other contemporary populations [34].

The teeth and jaws were examined for dental caries, periapical lesions, periodontitis and dental hypoplasia. 50% of the 24 adult individuals evaluated had caries, with males and females equally affected. The teeth of the subadults were caries-free. The intensity (43 lesions) of the caries was 6.8% of the evaluated adult teeth (n = 635). Intra-vital tooth loss (56 teeth) was 6.3% amongst the adults and if it is included, the intensity increases to 14.3%, which is actually low in comparison to other early medieval populations [35]. Six males and three females exhibited a total of 34 periapical lesions (osteitis/radicular cysts), resulting from dental caries. There was one case of dentogeneous sinusitis maxillaris. 17 of the 22 adults examined (85%) were affected by periodontitis. More than half of the individuals (22/39; 56.4%) revealed hypoplastic enamel defects, although children were less severely affected than adults. Overall, the dental health at Szolád was much better than at other early medieval sites [35], [36].

The cephalic dimensions of 16 adults from Szólád were recorded and used to determine length-breadth indices [37] (Table J in File S1). Long-headed cranial vaults predominated (Ind. 4, 11, 17, 22, 24, 27, 28, 43, 44). Three individuals were medium-headed (Ind. 3, 25, 45) and four were classified as short-headed. The latter included three females (Ind. 9, 19, 30) and a possible male (Ind. 5). According to Rösing [38] and Obertová [39] short-headed crania appear to be an exception at Szólád and perhaps confirm that the population did not originate in the locality.

Although no kinship analysis with a phenotypic approach was carried out by systematically recording anatomical variants of the skeletons and teeth, the investigation revealed a number of rare dental traits in certain individuals. These were independent of the genetic evidence for biological kinship (cf. Chapter 5.2). Two traits are especially noteworthy. They are double-rooted canines in the mandible identified in three individuals (Ind. 20, 30, 31) and deciduous canines remaining in the maxilla combined with maleruption of the permanent canines in individuals 14 and 37. The relevance of anatomical variants and anomalies in recognising biological kinship between several persons has been highlighted in numerous studies (cf. [40], [41]). This is usually investigated by systematically screening various anatomical variants and carrying out a biostatistical evaluation. At Szólád, the individuals with double-rooted teeth may have been blood-related, because the frequency of this trait exceeds what would usually be expected in a population [42].

### Molecular genetics

Overall, the mitochondrial DNA (mt-DNA) profiles of 28 out of 38 analysed individuals were successfully replicated (73.7%) (Table L in File S1) and the sequence data are deposited in GenBank (http://www.ncbi.nlm.nih.gov/genbank/; accession numbers KM114982 to KM115015). The remaining ten individuals showed insufficient DNA preservation (Ind. 16, 20, 26, 29, 31, 34, 35, 44) or yielded inconsistent results between samples A and B of the same skeleton (Ind. 6 and 21). The reproduced data of 28 individuals exhibited a high variability of mitochondrial haplotypes (78.6%). Twenty-two different lineages were identified that belonged to the haplogroups (hg) H (n = 9; 32.1%), U, J (both n = 4; 14.3%), HV, T2, I, N1b, K (each n = 2; 7.1%), and N1a (n = 1; 3.6%). This composition includes a large number of hgs that commonly occur in present-day European populations [43], while N1a and N1b are very rare in Europe today and increase in frequency towards the Near East [44], [45]. The detected frequency of hg H at Szólád was lower than it is in modern European populations (c. 45%) but higher than it is in the Near East and Caucasus today (c. 20%) [43], [46]–[48]. Hgs U4 and J at Szólád occurred twice or three times as often as in present-day Europe, while the frequencies of K and T were very similar to modern rates. Haplogroup HV makes up approximately 2% in extant north-western and northern European populations and increases to ca. 3.5% in south-eastern Europe and in the Near East [43]. Haplogroup I is most abundant in Scandinavia and north-western Europe, where it represents approximately 2.5% of the population, while it decreases to 1.5% towards southern and south-eastern Europe and the Near East [45]. Overall, the phylogeographical comparison revealed a highly variable composition of maternally inherited haplogroups characterised by both European and Near Eastern elements [43], [49] and matched the pan-European context of the study. Thus, the variability and the supra-regional parallels of the haplogroups identified highlight the heterogeneous composition of the burial community of Szólád.

Four haplotypes occurred in more than one individual, suggesting possible maternal kinship among the members of the burial community (Table L in File S1). Three of these lineages were further investigated by HVS-II sequencing to increase the phylogenetic resolution. Individuals 4, 18, 30, and 32 shared a common haplogroup H lineage, with an HVS-I sequence that corresponds to the rCRS (revised Cambridge Reference Sequence). Kinship among these individuals cannot be excluded, but seems rather unlikely since this haplotype is very common in modern-day European populations. For the three remaining haplotypes, each of which occurred in two individuals, maternal kinship seems to be more probable – at least if the rare occurrence of these haplotypes both nowadays and in earlier time periods is considered a compelling argument. To our knowledge no genetic data are available which would represent the time range and area investigated as part of our study, so that data from different time periods and European regions (in-house database) were used to calculate the abundance of identical haplotypes. In addition, the haplotypes of 27 Hungarian individuals from the 10^th^ and 11^th^ centuries [50] had no parallels to the three identical haplotypes found at Szólád. Many more data from contemporaneous burial communities are required to validate this assessment. Individuals 11 (m, 35–45 y.) and 42 (sex indet., 4–8 y.) shared an identical HVS-I and HVS-II lineage that belongs to hg K. This specific haplotype is found with a frequency of 0.62% in 21,724 sequences of present-day populations throughout Europe and the Near East (in-house database). Furthermore, an I3 haplotype was identified in individuals 14 (sex indet., 13–17 y.) and 8 (sex indet., 3–5 y.), which nowadays occurs at a rate of 0.027%. Individuals 13 (m, 35–50 y.) and 22 (m, 40–50 y.) shared the same lineage of haplogroup N1b2, which only has twelve equivalents (0.055%) among the modern-day reference data. Thus, these three pairs of relatively rare haplotypes (K, I3, N1b2) may perhaps reflect kinship relations within or between generations. Individuals 14 and 8 as well as 13 and 22 may have been siblings, while for individuals 11 and 42 maternal relations such as uncle and nephew or cousins are possible, considering the time span during which the cemetery was in use. Yet, the occurrence of identical haplotypes by chance cannot be excluded entirely, although it can be viewed as unlikely due to the archaeological context and the low frequencies among modern-day populations.

### Dietary reconstruction

**Figure 3:**
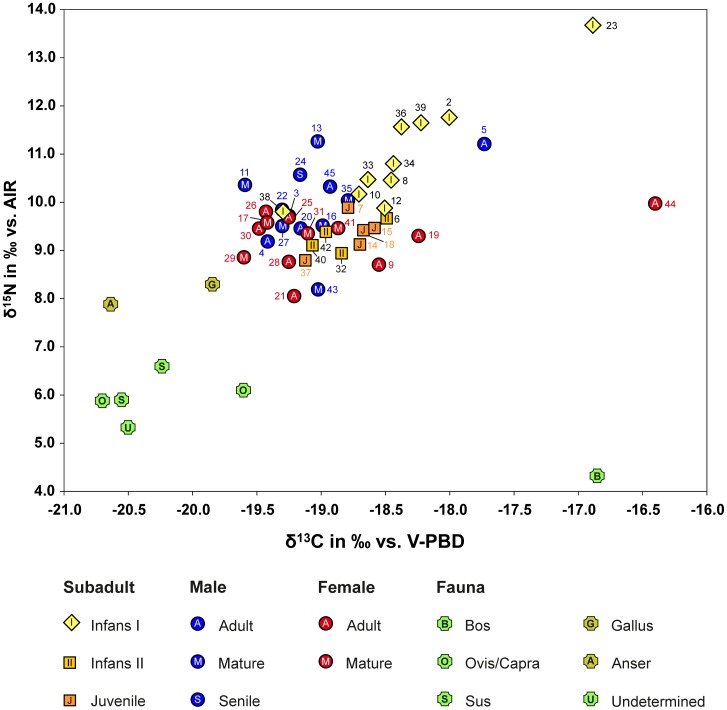
Scatter plot of δ^13^C and δ^15^N values of human and faunal collagen from the cemetery at Szólád (graphics: C. Knipper).

The human and faunal samples yielded well-preserved collagen that fulfilled the quality criteria of C and N content and atomic C/N ratios of archaeologically preserved collagen [51]. Acknowledging the considerable loss of material by ultrafiltration [52], samples with between 0.5 and 1% of collagen were included in the data evaluation (Table M in File S1). The domestic animals yielded δ^13^C values of between −20.7 ‰ and −19.6 ‰ vs. V-PBD (average: −20.3±0.4 ‰) (Table O in File S1; Fig. 3), which are typical of fodder mainly based on C_3_ plants. Only the δ^13^C value of −16.9 ‰ in one bovine (grave 5) reflects increased amounts of C_4_ plants, such as millet, amaranth and possibly sedges that may have been available on the shores of Lake Balaton. The difference in δ^15^N values between fowl (cock: 8.3 ‰ vs. AIR and goose 7.9 ‰) and domestic sheep/goats, pigs and cattle (average: 5.7±0.8 ‰), reflect different ratios of plant and animal protein in the animals' nutrition in accordance with their trophic levels.

**Figure 4:**
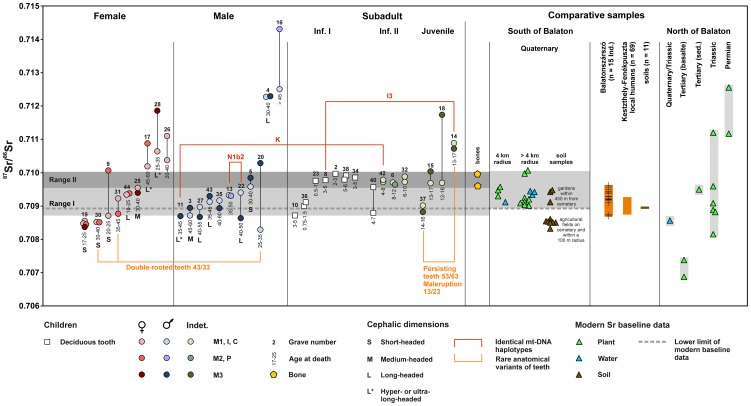
^87^Sr/^86^Sr ratios of human tooth enamel and bone from the cemetery at Szólád in comparison to modern vegetation, water, and soil samples from localities within a 12 km radius and from major geological units in the hills north of Lake Balaton. Ranges I and II are derived from the data distribution of the tooth enamel of the children, illustrated in the kernel density plot (Figure 5). The data from Kestzthely-Fenékpuszta and Balatonszárszó were taken from [79] and [57] respectively (graphics: C. Knipper).

The δ^13^C values of the human remains vary between −19.6 ‰ and −16.4 ‰. Individuals 44 and 23 (female, 18–25 y. and infans I, 0.5–1 y.) diverge from the main data cluster (δ^13^C = −19.6 to −17.7 ‰) by exhibiting higher δ^13^C values of above −17 ‰, which point to a significant role played by a C_4_ plant component in their diet (Table M in File S1; Fig. 3). Excluding individual 44, the average δ^13^C value of the adults was −19.1±0.4 ‰, which was 1.2 ‰ above the mean δ^13^C value yielded by the domestic animals. This difference is in agreement with the characteristic trophic level enrichment of the heavier carbon isotope [53], [54]. The average δ^13^C value of the adults from Szólád was up to 1 ‰ higher than that of Migration Period cemeteries in Germany and Austria (cf. data compilation in [[55], Fig. 4]). This may reflect the drier growing season at Lake Balaton in comparison to several of the central European sites (cf. [56]) or some impact of C_4_ plants on the diet of the population buried at Szólád. The latter hypothesis is also supported by higher average δ^13^C values compared to the Neolithic dataset from Balatonszárszó-Kis-erdei-dűlő located some 3–4 km from the Lombard period cemetery [57].

The nitrogen isotope ratios of the human bone collagen are closely comparable to those identified at other Migration Period cemeteries (cf. [55], Fig. 4 with further references) and reflect a mixed diet of plant and animal proteins. The average δ^15^N value of the adults was 9.6±0.8 ‰ which was 3.9 ‰ higher than the average of the domestic mammals and 1.5 ‰ above the domestic fowl. Inclusion of the age classes infans II and juvenile only marginally changes these values. Considering an enrichment of 3 to 5 ‰ per trophic level in the food chain [58], these data suggest that the human diet contained significant amounts of herbivore protein. Furthermore, food components with higher δ^15^N baseline values, such as poultry, meat of infant animals, or cereals that were grown on manured fields [59]–[61] may have contributed to the enrichment of ^15^N in human bone collagen. Given the location of the cemetery near Lake Balaton and the fact that at least in one case fish was placed in a grave as an offering (grave 27), freshwater fish was also considered as a potential food source with elevated δ^15^N values. Unfortunately, collagen extraction from Lombard period fish bones was unsuccessful, but analyses carried out on modern fish muscle tissue (which resembled the potentially edible component of the animals) from wetland areas near Lake Balaton produced δ^13^C values between −31 and −28 ‰ [62]. Even though the δ^13^C of freshwater fish can vary [63], the modern data are in agreement with a general trend towards a ^13^C depletion in their tissues [64]–[66], which would have lowered the δ^13^C values of the human bone. Therefore the relatively high δ^13^C-values measured in the human remains rather argue against the consumption of considerable amounts of freshwater fish, unless it was combined with C_4_ plants such as millet. Very similar C and N isotope ratios found in the ribs and long bones of three males who may have moved to Szólád (Ind. 3, 4, 22; cf. the discussion of Sr isotope data below) also refute the idea that their fish consumption would have increased after occupying the shores of Lake Balaton. Such a dietary change would have resulted in increased δ^15^N and decreased δ^13^C values in ribs, given that they remodel faster than long bones.

Among the age class infans I, some children aged up to three years (Ind. 2, 36 and 39) revealed distinct nursing signals [67]–[69], while other children aged two to three years (Ind. 8, 10, 34, 38) were in the process or had already been weaned. A child of no more than two months (Ind. 33) died too soon after birth to manifest a distinct nursing signal. In contrast, the stable isotope ratios of individual 23 (0.5–1 y.; δ^13^C = −16.9 ‰; δ^15^N = 13.7 ‰) exceeded those measured in the other children to a remarkable extent, suggesting that the mother would have yielded highly elevated δ^13^C and δ^15^N values, similar to the data gathered in male individual 5 who yielded unusual values compared to the other adults. However, the dataset lacks a possible breastfeeding mother or wet nurse with such isotope data ranges. In addition, starvation or a metabolic disease may also have contributed to the unusual stable isotope ratios of the infant [70], [71].

With a standard deviation of 0.8 ‰ (1 SD), the δ^15^N values of the adults were comparatively heterogeneous (cf. [55], [72]–[75]), which at least partly results from gender-specific access to animal protein. Six males (Ind. 5, 11, 13, 24, 35, and 45) exhibit δ^15^N values above 10.0 ‰ which do not occur among the females, older children and juveniles. This means that the males (mean δ^15^N = 9.9±0.8 ‰, n = 13) had significantly higher δ^15^N values than the females (mean δ^15^N = 9.2±0.6 ‰, n = 12; Student's t-test for homogeneous variances p = 0.024, Kolmogorov-Smirnov-test for normal distribution). The group of males with elevated δ^15^N values included the oldest man (Ind. 24) and individual 13 who was buried in the deepest and most elaborately furnished grave. The male individual 5 was remarkable in terms of his relatively high collagen δ^13^C (−17.7 ‰) and δ^15^N values (11.2 ‰), which may reflect larger shares of meat from omnivore or infant animals, or foreign origin from a locality with steady access to marine food sources or elevated baseline δ^13^C and δ^15^N values as have been observed, for instance, in the Eurasian steppes [76], [77]. Notably, a cattle bone found in the same grave also produced an elevated δ^13^C value of −16.9 ‰. Despite disturbances by grave robbers observed in many burials, grave furnishings and access to animal protein seem to correlate, especially among the males. This distinguishes them from the females, among whom for instance, individual 21 yielded the most elaborately furnished grave and the lowest collagen δ^15^N value.

Individuals 5 (adult male), 23 (infant), and 44 (adult female), distinguished by their elevated δ^13^C and partly also their δ^15^N values, were buried in different parts of the cemetery, including the centre (Ind. 5), the northern periphery (Ind. 23) and an isolated location some 30 m south of the other graves (Ind. 44). While individual 23 can be viewed as belonging to the main cluster of burials, the separate position of individual 44 is remarkable. Despite the interment of the body in a ledge grave as was typical of the Lombard period, the complete absence of grave goods is unusual. Although a chronological distinction cannot be entirely excluded, all these characteristics point to some sort of social distinction of the individual, even though spatial separation of individual graves is not uncommon in Migration Period cemeteries.

### Mobility patterns

#### Strontium isotope ratios of environmental samples

The vegetation samples collected around Szólád (south of Lake Balaton, localities 13–26; cf. Figure A in File S1) yielded strontium isotope ratios of between 0.70902 and 0.71004 (mean 0.70937±0.00072, 2 σ; n = 11). Within this range, tree leaves and ground vegetation from locality 18 (Table N in File S1) produced ^87^Sr/^86^Sr ratios of 0.71004 and 0.70998 respectively, while the ground vegetation from nine localities and four water samples exhibited ratios of between 0.70901 and 0.70958 (Fig. 4). A comparable range was found in leachates from garden soils at an approximate distance of 450 m from the cemetery (^87^Sr/^86^Sr = 0.70912–0.70948; SzB2, SzB4, SzB5). On the other hand, soil leachates from agricultural fields above the site or within a radius of up to 100 m yielded less radiogenic strontium with very similar ^87^Sr/^86^Sr values of between 0.70850 and 0.70855 (soil A – E; SzB1, SzB3; n = 7). Two human bones from the cemetery exhibited ^87^Sr/^86^Sr ratios of 0.70995 (Ind. 4) and 0.70957 (Ind. 17) respectively.

Overall, the vegetation, water, and bone data were consistent with the isotope ratios of biologically available strontium in the Pannonian Basin [78], human enamel data from the Neolithic site of Balatonszárszó-Kis-erdei-dűlő some 3–4 km from Szólád [57] and Roman to Carolingian period burials at Keszthely-Fenékpuszta on the western shore of Lake Balaton [79]. They were also closely comparable to data from other European loess landscapes that typically range between about 0.7090 and 0.7100–0.7110 [80]–[83]. Given the consistent offset towards Sr isotope values of common fertilizers, the relatively low and very uniform Sr isotope values of the soil leachates from agricultural fields (0.7085±0.0002 (2 SD)) are likely to have been influenced by fertilizers [[83], [84] (∼0.7084±0.0010 (2 S.D.))], and were therefore not given any consideration in the interpretation of the data gathered from the human remains.

The reference samples of vegetation from the Bakony hills north of Lake Balaton yielded more variable ^87^Sr/^86^Sr ratios which reflect the heterogeneous geology of the area. With one exception (locality 9), the vegetation collected on Triassic deposits provided strontium isotope ratios of between 0.70816 and 0.70960 (mean 0.70891±0.00104, 2 S.D.; n = 5). Tertiary basaltic deposits yielded the lowest bioavailable ^87^Sr/^86^Sr (0.70688 and 0.70738) ratios whereas the highest end-member was represented by the Permian units (0.71117 and 0.71255).

Taken together, the comparative vegetation, water, soil and bone data suggest that the ‘local’ Sr isotope signal for Szólád lies within the range of between about 0.7090 and 0.7100, although these values are discussed below in view of the peculiar Sr isotope data gained from the human remains.

#### ^87^Sr/^86^Sr isotope ratios of the human remains

**Figure 5:**
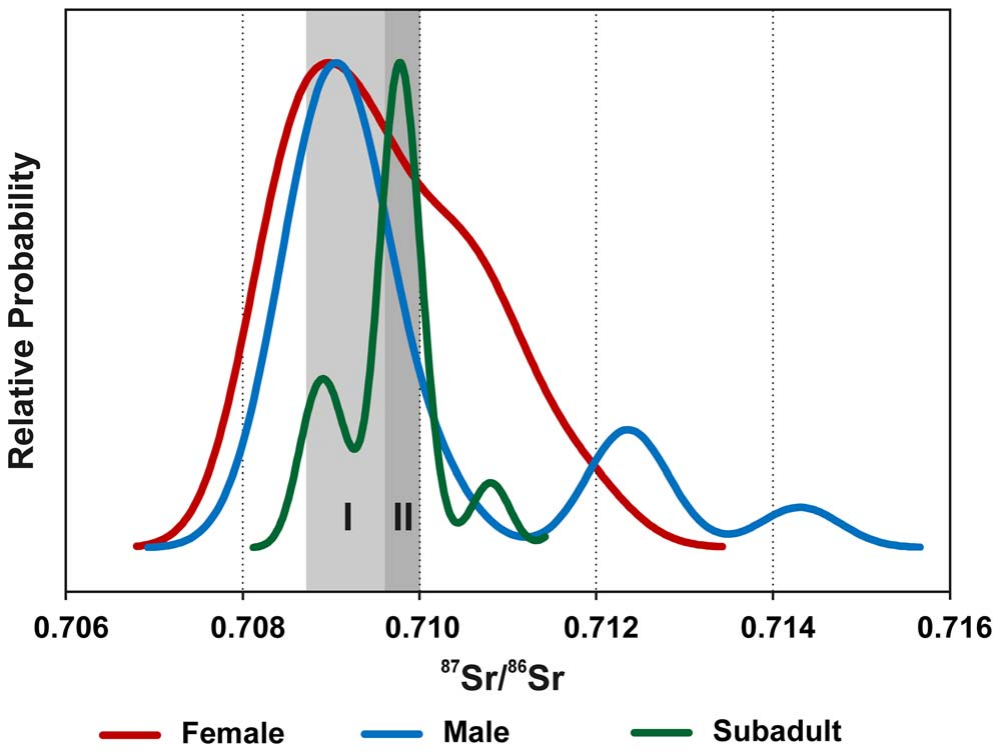
Kernel density plot of the ^87^Sr/^86^Sr ratios of the enamel of male and female adults and subadult individuals from the Lombard cemetery at Szólád (graphics: C. Knipper).

Given the problematic preservation of in-vivo Sr isotope values in bone [85], [86] we focused on tooth enamel of early and late mineralising teeth (largely second incisors/canines vs. third molars) for the purposes of this study, with a few exceptions for comparison. The corresponding ^87^Sr/^86^Sr ratios of all 66 enamel samples from 35 individuals ranged between 0.70829 and 0.71431 and showed a peculiar distribution between females, males and subadults (Figs. 4 and 5). We will focus on this pattern below and attempt to present an interpretative model. The data range observed was considerably wider than that measured at the nearby Neolithic site of Balatonszárszó-Kis-erdei-dűlő [57], which suggests the presence of first-generation arrivals from different locations.

The tooth pairs of six of the 35 Lombard individuals investigated (17.1%; adult female: Ind. 17, 26, 28; adult male: Ind. 4, 16; juvenile sex indet.: Ind. 14) yielded ^87^Sr/^86^Sr ratios above 0.7100. These values are more radiogenic than the comparative data from the area south of Lake Balaton. The early mineralising incisors, canines and first molars, in particular, indicate different non-local birthplaces and varying trends among males and females (Fig. 4). Three additional individuals (8.6%; adult female: Ind. 9; adult male: Ind. 20; juvenile sex indet.: Ind. 18) exhibited large differences in their Sr isotope values between their early and late forming teeth (>0.0012; defined as Δ(^87^Sr/^86^Sr)_early-late_/2 SD_SRM987_>70), and the same phenomenon was observed in individuals 16 and 28 mentioned above. Given that the offsets between Sr isotope ratios of teeth from the same individual otherwise tend to be rather small, the data here point to residential changes during childhood. A closer inspection of these individuals reveals that in at least three cases (Ind. 16, 18, 28) the late mineralising teeth yielded more clearly non-local values than the corresponding early teeth. Individual 18, who died at the age of 12–16, is particularly interesting, with a third molar that was clearly non-local (0.7117), whilst the second incisor had a Szólád-compatible ^87^Sr/^86^Sr ratio of 0.7097. This suggests that this person must have died at Szólád very soon after their arrival. Otherwise the molar, which was probably still developing (given the age at death of 12–16 y.) would have quickly become overprinted with the less radiogenic Szólád soil signature. All this points to a highly mobile community.

Owing to the rather consistent spread of data with regard to both female and male individuals and a residual uncertainty in terms of the range of the comparative baseline values presented above, we are less certain with regard to identifying non-local individuals at the non-radiogenic end of the human data distribution. However, all analysed teeth of females 19 and 30 exhibited ^87^Sr/^86^Sr ratios below those of the modern comparative data and the enamel ratios of human remains from archaeological sites on Lake Balaton [57], [79]. They also lie below those of the children, a section of the burial community that in other studies has repeatedly been shown to exhibit the smallest variation [cf. 87, 88]. If individuals 19 and 30 are considered to have been non-local, at least 31.4% of the burial community (11 of 35) would have died at a location other than their birthplace and/or would have moved during childhood (^87^Sr/^86^Sr_Szólád_ 0.7090–0.7100). Because ^87^Sr/^86^Sr ratios of between 0.7090 and 0.7100 are typical of loess landscapes in general and are thus very common outside of our study area [80]–[83], intra-loess mobility cannot be identified and non-local individuals may actually exceed 31%.

In addition to the identification of outliers, the data distribution as illustrated in a kernel density plot discloses very revealing age and sex-specific patterns (Fig. 5). Depending on the data availability, this analysis includes all data obtained from pairs of early and late forming teeth or single teeth per individual. In order to avoid female individual 19 being overrepresented on account of the fact that four of her teeth were analysed, only the data of one incisor (tooth 32) and of the third molar (tooth 28) were included.

The density curves exhibit a narrower major mode for adult males than for females. The children have a minor mode (∼0.7089) that matches the major mode of the males, while a very narrow peak of more radiogenic Sr isotope ratios represents the majority of the subadults (∼0.7098). We defined ranges I and II based solely on these two modes exhibited by the children's data. Range I ranges from 0.7087 (Ind. 10) to the lower limit of range II, which in turn is indicated by ^87^Sr/^86^Sr ratios of between 0.7096 (Ind. 40) and 0.7100 (Ind. 15), coinciding with the maximum value of the modern vegetation samples taken in the area. Referring to our reference samples and published strontium isotope data for the region, all ^87^Sr/^86^Sr ratios within the two ranges could have resulted from the consumption of food/water originating from the surroundings of the cemetery, i.e. the region south of Lake Balaton. It seems unlikely, however, that the virtual absence of ^87^Sr/^86^Sr ratios of range II among the adults, which was the best-represented amongst the children (73%; 11 of 15) was due to random chance.

#### Three-phase model of the settlement history of Szólád

**Figure 6:**
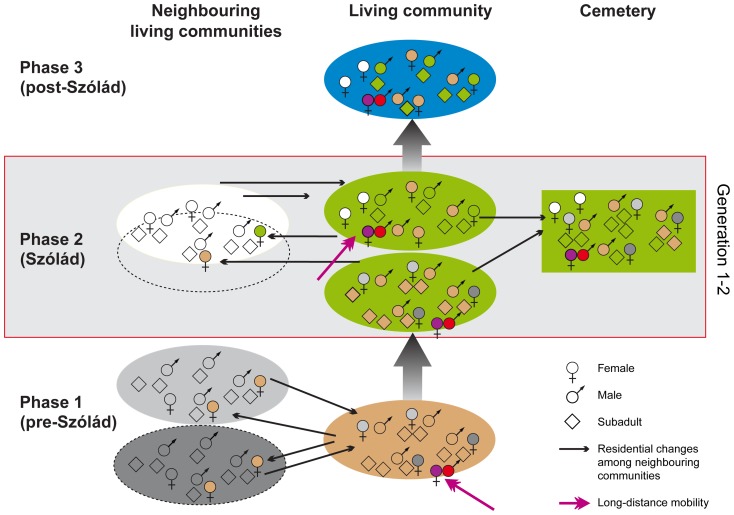
Model of the Szólád community's residential changes and occupation of the cemetery. The background colours symbolise the different Sr isotope baseline values of the settlement locations. The symbols for the females, males and subadults are marked with the colour of the locality of their childhood. The colours red and purple denote long-distance migrants. Individuals with shades that differ from the background are non-local to the locations they are mapped in. The model is a hypothetical explanation of the occupation of the Szólád cemetery with local and non-local individuals in line with the strontium isotope data distribution (graphics: D. Peters, C. Knipper).

Given the historical context of the Migration Period, the occupation of Pannonia by the Lombards, the archaeological evidence suggesting a very short period of occupation of the site (c. 20 years: [8], [13]), and the indication of different birthplaces of adults and children allows us to propose a three-phased model of residential change and group movement (Fig. 6). We consider this a plausible explanation for the ^87^Sr/^86^Sr ratio distribution observed amongst age/sex-defined sub-groups of the Szólád burial community, and will address uncertainties concerning the local baseline values and alternative interpretations below:

*Phase 1 (pre-Szólád)*: The first phase of group movement represents a community residing at a location with biologically available strontium characterised by Sr isotope ratios in range I (0.7087–0.7096). Such values are also quite common in the vicinity of Szólád and thus do not allow us to infer the distance of movement. The minor mode of the Sr isotope data of the children very closely matches the major mode of the males, whilst the females exhibit a considerably wider data distribution. This implies movement of women into the community of the men, hence patrilocal residential rules [81], [89], [90]. Children that were perhaps born in this location (Ind. 10, 36, 37, and 40) have Sr isotope ratios similar to those of their potential fathers, but not necessarily of their mothers.

*Phase 2 (Szólád)*: At least some families – if not the entire community – abandoned their place of residence and moved to Szólád. Some of the children with ^87^Sr/^86^Sr ratios in range I (Ind. 10, 36, 37, and 40) did not reach adulthood and were buried in the newly established cemetery. At the same time the community had offspring at the new location, although child mortality rates were high. The larger number of children in Sr isotope range II implies that they represent offspring who were born at Szólád and died during their first years of life (infans I and II). Their ^87^Sr/^86^Sr ratios (0.7096–0.7100) represent the biologically available strontium in the plots that were farmed by the Szólád community. During the period the cemetery was in use more adults with Sr isotope ratios in range I or with more variable ratios indicating different birthplaces died and were buried at Szólád.

*Phase 3 (post-Szólád)*: The virtual absence of adults with Sr isotope ratios in range II, which contains the majority of children, suggests that the settlement and cemetery were abandoned after less than one generation, i.e. approximately 20 years. Individual 5 (m, 30–40 y.) and individual 9 (f, 20–25 y.) were the only adults with one or both ^87^Sr/^86^Sr ratios within range II. However, the markedly different δ^13^C and δ^15^N values of the bone collagen of individual 5 raise the question whether this was the oldest representative of those individuals who grew up at Szólád. The individual may also have been of non-local origin, however, from a locality characterised by similar Sr isotope values. Individual 9 exhibited a pronounced shift from ^87^Sr/^86^Sr of 0.70870 in her second incisor to 0.71007 in her second molar, which may indicate that she witnessed the residential change of her community to Szólád at some stage during her childhood. The evidence suggesting that the cemetery and associated settlement were occupied only for a very short period of time is in agreement with the historical records attesting to a short phase of Lombard settlement in Pannonia [5], [6] on one hand and with the limited chronological range of the grave goods on the other [8], [13]. The people who were born at Szólád and reached adulthood moved away and died elsewhere and are therefore not represented by our sample.

*Limitations and uncertainties regarding the settlement history model of Szólád*: The settlement history model of Szólád relies more on the Sr isotope data distribution in the human dental enamel than on the reference samples. The main arguments for the assumption that the population had moved there are the different 87Sr/86Sr ratios of adults and subadults as well as the non-continuous Sr isotope distribution within the category of children (ranges I and II). If a single sedentary community can be expected to display normally distributed ^87^Sr/^86^Sr ratios [87], the presence of different data ranges suggests diverging birth locations. The number of children with Sr isotope ratios in range II and the agreement of this range with the two human bone ratios, viewed as representing the isotope ratios of the labile strontium available at the site [91], allows us to interpret the data in range II as attesting to people who consumed resources grown near the cemetery in their childhood.

However, extant plant and water samples taken from a radius of some 12 km around Szólád predominantly yielded ^87^Sr/^86^Sr isotope ratios of between 0.7091 and 0.7095. These values match range I of the tooth enamel data of the children, which is considered by the model to reflect a location other than Szólád. Environmental data in range II are underrepresented. This apparent discrepancy raises questions regarding the spatial resolution of the reference data and their suitability as baseline samples. In trying to avoid modern-day fertilised fields and vineyards, we may have selected sample locations that did not actually include the areas that were farmed by the Szólád community. Furthermore, despite sampling modern forests we cannot exclude the possibility that slight shifts took place in the Sr baseline values over time due to decalcification of the soils and/or anthropogenic atmospheric input [83], [92], [93].

The ^87^Sr/^86^Sr ratios of the human enamel from Balatonszárszó (0.70868 and 0.70962) partially coincide with both isotope ranges at Szólád and do not cover range II completely [57]. This may have been caused by minor variations in the isotope composition of the strontium biologically available from the loess south of Lake Balaton.

Even though range I is in better agreement with the reference data from around Szólád, the alternative idea that range I may represent the local values is less convincing if one considers the age and sex distribution of the individuals that yielded values within this range. This would imply a much higher number of non-local children with many local adults, a pattern, which would be difficult to explain in terms of settlement dynamics. It also contradicts the archaeological and historical evidence suggesting a short period of occupation.

Overall, our model is a hypothesis that goes beyond the identification of single non-local individuals within burial communities and uses strontium isotopes to resolve group residential dynamics with regard to a time period characterised by the historical sources as profoundly shaped by human mobility. However, due to small-scale regional variations of the isotope ratios of biologically available strontium and multiple occurrences of similar data spectra, the existing Sr isotope data alone do not allow us to infer the distances travelled by the group. A small-scale shift in the location of the settlement and farmland is as plausible as a movement covering a greater distance of several dozens of kilometres.

## Data Integration and Concluding Interpretation

The archaeological evidence gathered from a large number of well-armed males and equally richly equipped females suggests that the cemetery at Szólád was a burial ground for various contemporaneous small groups *(Personenverbände)* of equal social status with their own internal hierarchies. In contrast to biological families, the historical term *familia* refers to social groups of both related and unrelated members of households. The term *fara*, which was only used for 8^th^ century Lombards, includes both kinship relations and group contexts during periods of resettlement/migration ([24], p. 187).

The results of the interdisciplinary analysis of the archaeological and anthropological data support the hypothesis that the population moved as a group whilst also integrating unaffiliated individuals, as indicated by ^87^Sr/^86^Sr ratios outside of ranges I and II (Figs. 4 and 5). This unravels biological ties and reflects local, regional and interregional relations as well as heterogeneity concerning cultural and biological properties.

Our results support the historical tradition of a movement of people during the Migration Period. The grave goods do not show any chronological differentiation within the cemetery and the specific distribution of strontium isotope data suggests that the adults grew up at localities other than those of many of the children. Shared mtDNA haplotypes are rare and indicate only a small number of deaths among members of the same families during the use of the cemetery. This is to be expected if the site was only occupied for some 20 years because the shorter the period during which a burial ground was used, the less likely we are to find genetically related individuals. The pairs of individuals who do share rare haplotypes (13 and 22; 14 and 8; 11 and 27) were deposited to the north and south of each other only some 10 m apart (Figure S1). Other remarkable features are circular ditches surrounding graves 14 and 8, while no such features were recorded for graves 11 and 42. Grave 13 was enclosed by a ditch, while grave 22 was not. Overall, the locations of the graves of individuals that may have been maternally related suggest that kinship and close social ties typical of *familiae* were respected in the spatial organisation of the cemetery and may have played a major role in the appearance of the typical row-grave cemeteries of the Merovingian period in central Europe.

Some of the individuals with the same maternal lines or rare anatomical variants have Sr isotope ratios in different data ranges (Fig. 4; Figure S1). For instance, individual 11 (m, 35–45 y.) with Sr isotope ratios in range I has the same mtDNA haplotype (K) as individual 42 (4–8 y.) with ^87^Sr/^86^Sr ratios in range II. Equally, individual 8 (3–5 y., range II) shares the I3 haplotype with the non-local individual 14 (13–17 y). These connections and the age and sex-specific distribution of Sr isotope ranges mirror the settlement history of the community and suggest that the representatives of both isotope ranges belonged to the same living population that changed its place of residence as a group. Nevertheless, one must keep in mind that the isotope ratios of range I, which our model interprets as having originated from the place of the community's former residence, are generally very common in the loess landscape south of Lake Balaton and elsewhere in central Europe. Therefore, no conclusions can be drawn with regard to the distance the group travelled. Based on the strontium isotope data alone, it is in this case impossible to distinguish between small-scale resettlement within only a few kilometres and long-distance migration. Overall, the results of our bioarchaeometric study of the Szólád cemetery, particularly the lack of range II adults amongst the burials, support the notion promoted by the historical evidence that the “Lombards” remained in Pannonia for a short period only. However, one must not view this as final confirmation of the historical statements until other burial communities from the 6^th^ century A.D. have been investigated and shown to reveal similar trends.

Moreover, the burial community is characterised by both biological and cultural heterogeneity. The individuals with Sr isotope ratios that deviate from ranges I and II (31.4% of the individuals analysed) suggest that people of diverse origin were integrated – in cases of highly radiogenic ^87^Sr/^86^Sr (>0.7120) ratios, most likely from beyond Pannonia – and that residential changes took place during some individuals' childhoods. Biological diversity is further reflected by the mtDNA haplogroup composition that includes numerous characteristic central European lineages and a few individuals with haplotypes that rarely occur among modern or prehistoric populations from this area. The aDNA data are in agreement with the isotopic evidence of patrilocality and higher mobility amongst women. Biological diversity is also supported by the apparent differences in the length-breadth indices of the cranial vaults. Especially noteworthy are three females (Ind. 9, 19, 30) with remarkably short head lengths and very similar strontium isotope ratios in their early formed teeth of around 0.7086±0.0001. Moreover, individuals 19 and 30 share very short body heights of about 154 cm and almost identical δ^15^N values of 9.3 and 9.5 ‰ respectively, while individuals 9 and 19 have above average δ^13^C values of −18.6 and −18.4 ‰.

Several rare anatomical variants observed in the teeth also point to biological ties, but are not in accordance with the genetic evidence for possible direct relations among pairs of individuals. The indications that the population was heterogeneous match the remarkable intra-group variation of the carbon and nitrogen stable isotope data of bone collagen, which reflects food consumption from diverse agricultural plots, and in some cases points to a potential consumption of millet and varying amounts of animal-based products. Sex-specific patterns point to social inequality as reflected in the men's preferred access to animal-derived foodstuffs. This is especially remarkable among the well-furnished burials, which probably represent higher social ranks. The collagen stable isotope data do not point to an extensive consumption of freshwater fish, which would have been readily available from Lake Balaton nearby. Whilst further confirmation could be obtained from sulphur isotope data [66], [94], this observation supports the notion that the community had not long arrived in the locality and suggests that they adhered to the sociocultural traditions that had previously been established in areas where fish was less readily available.

The child mortality rate of 22.7% exhibited by the age group infans I (0–6 y.) appears higher than that of contemporaneous cemeteries in south-western Germany [26], for instance, but may be a reflection of different burial customs. While the risk of contracting infection and complications generally increases at the end of the nursing period due to the introduction of solid food, many active periosteal lesions of children at Szólád point to additional chronic infectious and deficiency diseases or anaemia which led to a number of infant deaths. Because these findings primarily occur among children with Sr isotope ratios in range II, who, we assume, were born at Szólád, the increased child mortality rate is unlikely to have been caused by a mobile lifestyle. Malnourishment, however, due to the fact that the farming economy had not yet been fully established in the new place of residence, could possibly explain the phenomenon. Both the rich offerings of weaponry in some of the male burials and the recurring evidence of interpersonal violence point to the difficult political situation during the Migration Period.

The biological evidence suggests that the residents of Szólád were not a close reproductive community. This is in agreement with the notion of a partnership of convenience that resembled Germanic tribe formations with people of different cultural backgrounds maintaining regular contact with other contemporary *gentes*. Influence from several different European regions is supported archaeologically by the grave constructions that included ledge graves and graves with straight walls, some of which were surrounded by rectangular or circular ditches. The stylistic analysis of the grave goods, such as brooches and weaponry, revealed parallels to south-western and central Germany, Moravia and the middle Danube as well as to Italy. The latter also indicates the possible presence of members of the Roman population of Pannonia, who had settled the area prior to the Lombard period. The precious metal artefacts and the production quality of many of the grave goods reflect the prosperity of the community and their long-distance contacts. Direct evidence for their active involvement in long-distance trade is provided by grave 13, which contained an equestrian with a weighing scales and weights as well as coins and Thuringian pottery [20].

In summary, the historical, archaeological and bioarchaeometric data suggest that the Szólád cemetery was in use for a short period of time only. The study has revealed an example of a community arriving in and departing from Pannonia, a region which served as a melting pot for various cultural traditions. Moreover, the interdisciplinary approach that involved an unprecedented array of techniques, including aDNA as well as stable and radiogenic isotope analyses, further illuminates the role of population dynamics during the European Migration Period.

## Supporting Information

Text S1 **Grave furnishing.**
(PDF)

Text S2 **Methods.** Osteology, Molecular genetics, Carbon and nitrogen isotope analysis, Strontium isotope analysis.
(PDF)

Text S3 **Results.** Osteology.
(PDF)

File S1 **Data tables.** This file includes Table A – Table O and Figure A. Table A, Individual-based skeletal profiles; Table B, Demographic distribution of the Szólád population; Table C, Frequency of adults and subadults in different Lombard cemeteries; Table D, Average body heights of different Lombard and Thuringian populations; Table E, Individuals from Szólàd with healed and perimortem injuries; Table F, Frequency of degenerative joint disease in adult individuals from Szólád; Table G, Individuals with endo- and ectocranial periosteal reactions; Table H, Individuals with postcranial periosteal reactions; Table I, Distribution of Cribra orbitalia in adults and subadults; Table J, Cephalic index (length-breadth) of individuals from Szólád; Table K, Primer sets used for PCR analysis; Table L, Analysed samples and results for HVS-I, HVS-II, and selected coding region SNPs for all analyzed individuals; Table M, Collagen yields, nitrogen and carbon contents, atomic C/N ratios, carbon and nitrogen isotope ratios of human collagen, and strontium isotope ratios of human teeth and bones from the cemetery of Szólád; Table N, Strontium isotope ratios of vegetation, water and soil samples; Table O, Collagen yields, nitrogen and carbon contents, atomic C/N ratios, and carbon and nitrogen isotope ratios of faunal collagen; Figure A, Sampling localities of vegetation and water samples for strontium isotope analysis.
(XLSX)

Figure S1 **Map of the cemetery of Szólád with indication of anthropological age and sex determinations and results of aDNA and strontium isotope analysis.**
(PDF)
