# Supplementary material for: Lombards on the Move – An Integrative Study of the Migration Period Cemetery at Szólád, Hungary
Source: PLoS One. 2014 Nov 4;9(11):e110793. doi: 10.1371/journal.pone.0110793 (PMC4219681; doi:10.1371/journal.pone.0110793)
Supplement: Text S1 — Grave furnishing. (PDF) [file pone.0110793.s001.pdf]

Text S1: Grave furnishing

Even though the restoration of the finds has not yet been completed, enormous richness of grave furnishing is already evident (Fig. 2; Fig S1 a-c). It includes clothing equipment, jewellery, weaponry as well as items of daily use and ceramic vessels. The very thorough excavation furthermore revealed many discolorations by organic grave goods, such as wooden vessels, which are usually lacking. Despite disturbance by grave robbery, many inhumations were wealthily equipped. Owing to the careful research excavation, it was in many cases even possible to identify objects which were stolen from the graves in ancient times. Items of weaponry in 84 % of the interments of adult and juvenile males indicate the relevance of a military component at the site. In correspondence to the large number of weapon graves, the furnishing of the women's graves with brooches is especially rich [1, p. 408]. Half of these interments (n = 7) revealed 16 fibulae. Precious items of costume, such as bow brooches, occurred exclusively in undisturbed, unusually deep and elaborately constructed graves. There they were combined with a pair of S-fibulae, which corresponds to fashion trends of the row grave civilization of the 6th century. Lombardian cemeteries in Pannonia, Moravia, Austria, Slovenia, and Italy revealed very similar grave furnishings for males and females, which enlightens contacts and ties among the areas which were postulated to have been crossed by the Lombardian Migration (e. g. S-brooches of the Varpalota type in grave 21 [2, p. 373, Anm. 52] cf. [3, plates 70, 4, 188, Fig. 13.B.3-5, 5]. The appearance of identical types in Merovingian (western) areas of the row grave civilization is usually interpreted as evidence of resettlement of the Lombards [6]. However, in the 6th century when mobility played a significant role, long distance contacts due to trade, war, and intermarriage, and independent from ethnically determined migrations are very likely. Moreover, other grave goods, such as ceramics, and the grave constructions point to strong ties towards the central German-Thuringian area (cf. [7, 8, p. 198-199, Fig. 10-11, p. 224-225] for Thuringian brooches and ceramics see also maps in [4, p. 195, Fig. 20.1-2]). Some artefact groups revealed very close similarities among Pannonian, Moravian and in some cases even central German finds which indicate production in identical workshops.

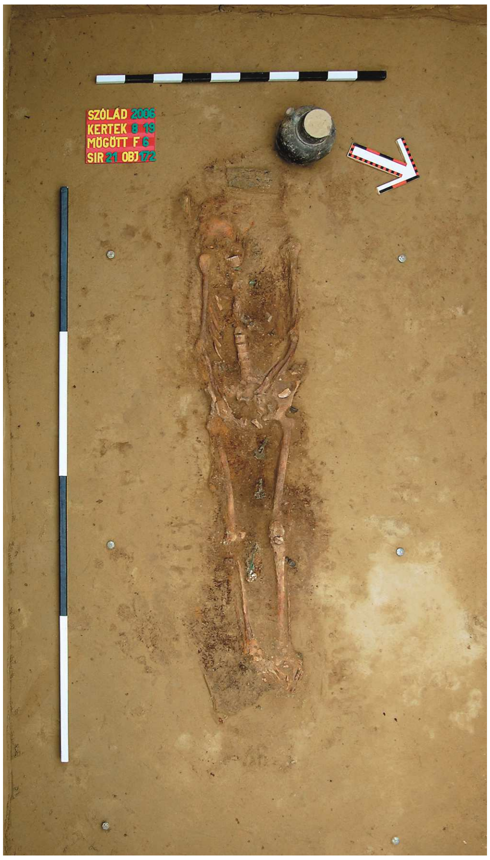

S1 a): Grave 21 (female) during the excavation (Photo: U. v. Freeden, T. Vida).

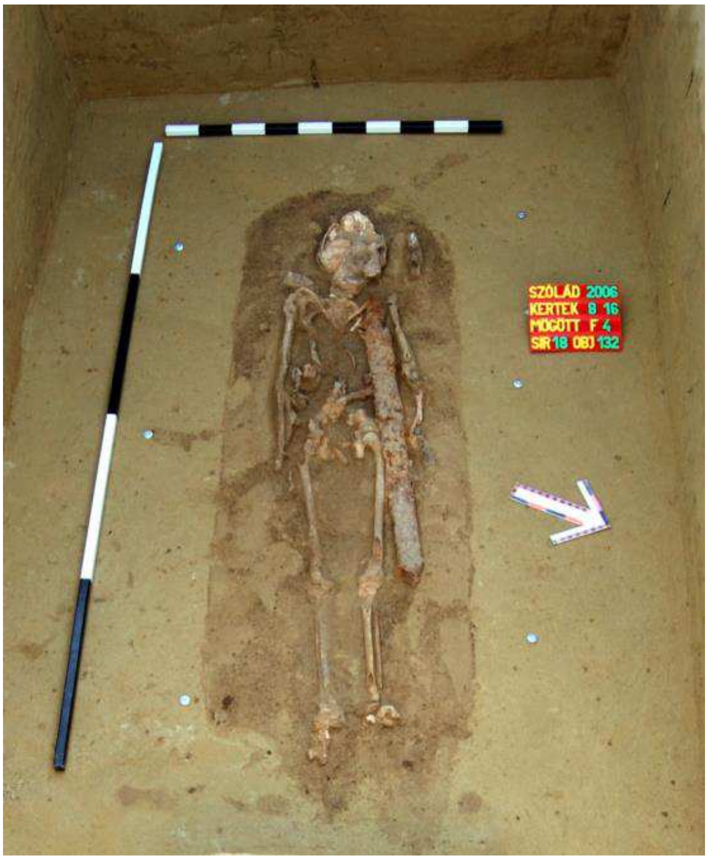

S1 b): Grave 18 (male) during the excavation (Photo: U. v. Freeden, T. Vida).

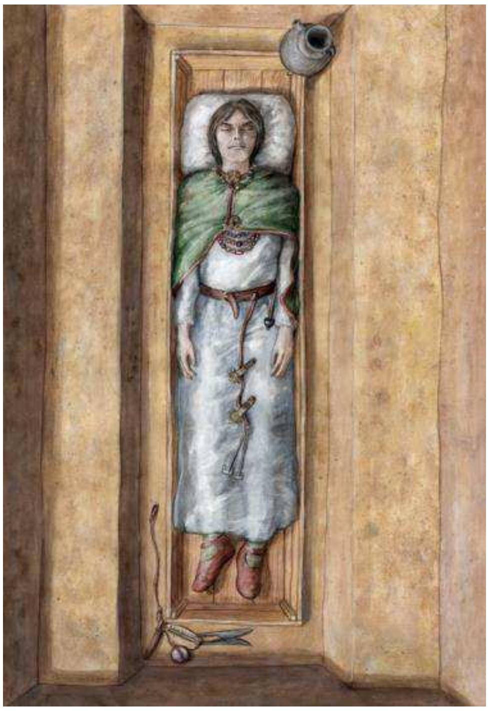

S1 c): Reconstruction of grave 21 (female). (drawing: F. Spangenberg, illu-atelier.de; LVR-Landesmuseum Bonn).

References

1. Freeden U von (2008) Ausgewählte Befunde aus dem langobardenzeitlichen Gräberfeld von Szólád, Komitat Somogy. In: Bemmann J, Schmauder M, editors. Kulturwandel in Mitteleuropa Langobarden – Awaren – Slawen. Akten der Internationalen Tagung in Bonn vom 25. bis 28. Februar 2008. Frankfurt a. M., Bonn: Habelt. pp. 399-413.
2. Freeden U von, Vida T (2007) Ausgrabung des langobardenzeitlichen Gräberfeldes von Szólád, Komitat Somogy, Ungarn. Germania 85: 359-384.
3. Werner J (1962) Die Langobarden in Pannonien. Beiträge zur Kenntnis der langobardischen Bodenfunde vor 568. München: Bayerische Akademie der Wissenschaften.
4. Tejral J (2005) Zur Unterscheidung des vorlangobardischen und elbgermanisch-langobardischen Nachlasses. In: Pohl W, Erhart P, editors. Die Langobarden Herrschaft und Identität. Wien: Verlag der Österreichischen Akademie der Wissenschaften. pp. 103-200.
5. Milavec T (2007) Prispevek h kronologiji S-fibul v Sloveniji. Arheološki vestnik 58: 333-355.
6. Graenert G (2000) Langobardinnen in Alamannien. Zur Interpretation mediterranen Sachgutes in südwestdeutschen Frauengräbern des ausgehenden 6. Jahrhunderts. Germania 78: 417-447.
7. Quast D (2010) Die Langobarden in Mähren und im nördlichen Niederösterreich – Ein Diskussionsbeitrag. In: Pohl W, Mehofer M, editors. Archaeology of Identity – Archäologie der Identität. Wien: VÖAW. pp. 93-110.
8. Steuer H (1982) Schlüsselpaare in frühgeschichtlichen Gräbern. Zur Deutung einer Amulett-Beigabe. Studien zur Sachsenforschung 3: 186-247.
