# Supplementary material for: Lombards on the Move – An Integrative Study of the Migration Period Cemetery at Szólád, Hungary
Source: PLoS One. 2014 Nov 4;9(11):e110793. doi: 10.1371/journal.pone.0110793 (PMC4219681; doi:10.1371/journal.pone.0110793)
Supplement: Text S2 — Methods. Osteology, Molecular genetics, Carbon and nitrogen isotope analysis, Strontium isotope analysis. (PDF) [file pone.0110793.s002.pdf]

Text S2: Methods

Osteology

The ages at death, sexes, body heights, diseases, traumata and stress markers were determined using standardised osteological methods [1]. The tooth development and the closure of the epiphyseal sutures provided the values underlying the age estimations of children and juveniles [2], while the aging criteria for adults were the closure of the ectocranial sutures [3], the structure of the facies auricularis [4], the relief of the symphysis of the os pubis [5], tooth abrasion [6], as well as the combined method proposed by Nemeskéri et al. [7]. The sex determination of juveniles and adults was based on morphometric features of the pelvis and the skull [8-11]. The sexes of the children were not determined. The body heights were calculated from long bone measurements using formulas proposed by Pearson [12]. The Pearson formulas were used because they were established based on populations that had not yet been affected by the increase of average body heights in the 20th and 21st centuries. Moreover, reference data from other studies of Lombard period cemeteries were available. Bone and tooth traits for morphological kinship analysis were determined according to [13] and [14]. The diagnosis of diseases, traumata, and stress markers followed pathological standards and methods [15-22]. Where necessary, imaging techniques such as radiography and computed tomography were applied.

In order to ensure a detailed data acquisition the skeletons were broken down into 140 sections, all of which were assessed. This allowed us to calculate the ratio of individuals or bone regions affected by disease or trauma in relation to the total number of observable sections [23]. Osteoarthritis was scored in four grades including slight (1), moderate (2) and severe (3) alterations as well as age-related or arthritis-induced ankylosis (4). Caries appearance was scored into four stages: caries superficialis (enamel affected), media (dentin affected), profunda (2/3 of dentin affected, pulpa aperta), and tooth destroyed [24].

Molecular genetics

Two to four tooth samples were taken from each of the 38 individuals under DNA-free conditions (S2, Tab. 12). The samples were analysed in the ancient DNA facility at the University of Mainz, taking the usual precautions against contamination and using the established authentication criteria. These included separate pre and post PCR laboratories, routine cleaning of all the rooms, work benches, laboratory items, and instruments with bleach and UV irradiation overnight, eliminating external contaminants on the sample surfaces by shot blasting as well as UV irradiation, detection of contamination during the analyses by extracting negative controls and PCR blanks, replication of the results by multiple independent DNA extraction and amplification as well as cloning and typing of the researchers. The genetic analyses focused on the determination of the mitochondrial DNA (mtDNA) diversity of the Szólád cemetery. Therefore, informative positions of the control and coding regions were analysed (S2, Tab. 11). These included the hyper variable segments I (HVS-I, nucleotide

positions 15997-16409) and II (HVS-II, nucleotide positions 34-397), and in some cases the haplogroup defining positions of the coding regions, such as 7028 (haplogroup H) and 12308 (haplogroup U). Sequence data were replicated by at least three independent amplifications from two different samples. In addition, selected PCR products were cloned and 5-8 clones per PCR were sequenced to monitor possible background contamination and DNA damage. The sample preparation, DNA extraction, amplification, cloning, and sequencing followed standard protocols as previously described [25-27]. Sequence polymorphisms (S2, Tab. 12) were reported relative to the revised Cambridge Reference Sequence (rCRS) [27] and haplogroup assignment was carried out using the software HaploGrep (http://haplogrep.uibk.ac.at/) [29] based on the mitochondrial haplogroup phylogeny of phylotree (http://www.phylotree.org/, built 14, accessed 5 Apr 2012) [30]. Shared haplotypes of the Szólád cemetery were compared to an in-house database of 21,724 published HVS-I and II sequences, with the aim of assessing kinship probabilities. Sequences have been deposited in GenBank (http://www.ncbi.nlm.nih.gov/genbank/; AccNo. KM114982-KM115015).

Carbon and nitrogen isotope analysis

Collagen extraction followed [31] and [32] with some modifications. Samples were cut, and the surfaces and adhering cancellous bone abraded with dental cutting and milling equipment. Approximately 200-300 mg of bone were demineralised in 10 ml of 0.5 N HCl at 4°C for 14 days, and neutralised in three or more rinses, followed by gelatinisation in 5 ml of acidified H_2O (pH 1-2) for 48 hours. The insoluble portion was separated using pre-rinsed MIDI filters, and the remaining solution transferred into Amicon© ultrafilters (Millipore; cutoff, <30 kDa) which concentrated the long-chained collagen molecules. Rinsing with deionised H_2O, 0.1 M of NaOH and three times with H_2O ensured complete elimination of the glycerol coating of the filters prior to use. The filtered collagen was dried and weighed, and then analysed in duplicates using an elemental analyser (vario EL III, Elementar Analysensysteme) coupled to an IsoPrime High Performance Stable Isotope Ratio Mass Spectrometer (GV Instruments). Isotope compositions were reported in δ notation in per mil relative to V-PDB for carbon and AIR for nitrogen. The raw data were normalised using two-point calibrations based on USGS 40 and IAEA N2 for nitrogen and CH6 and CH7 for carbon [33]. Reproducibility of internal and external standards (sulfanilic acid, USGS 40) was better than ± 0.2 ‰ for nitrogen and ± 0.1 ‰ for carbon.

Strontium isotope analysis

The teeth were sliced in half using a water-cooled, diamond-impregnated rotating blade of 0.3 mm thickness. Enamel samples were prepared from one half of each tooth using a rotating disc attached to a handheld drill; the remaining dentine was removed and finally all surfaces were cleaned using a dental burr. No dentine remained attached to the enamel, which was thoroughly checked under a binocular microscope. Enamel sample sizes prior to leaching ranged between 6.3 and 84 mg.

Overall, analytical protocols followed [34] with certain modifications: Prior to dissolution, enamel fragments were cleaned ultrasonically in reagent-grade acetone, methanol and repeatedly in deionized H_2O, and leached overnight with 1 ml of ~0.25 M acetic acid to remove adsorbed or least strongly bound and most easily diagenetically affected Sr [35]; leachates were not analysed. Subsequent dissolution (wet-ashing) was performed overnight in thoroughly acid-cleaned, closed 7 ml PFA vials using 1 ml of ~15 M HNO_3 for each vial. The dissolved samples were spiked with an ^{84}Sr tracer, and the Sr was separated using extraction chromatographic columns using 100 µl SrSpec resin, with H_2O and a final drop of 6 M HCl used as eluting agents for Sr. The Sr isotopic compositions (and Sr contents) were measured using a VG354 thermal-ionisation multi-collector mass-spectrometer (TIMS) at Royal Holloway University of London (RHUL), while several smaller samples were re-analysed using a Triton TIMS at The Open University (OU, Milton Keynes, UK), in both dynamic and static Faraday modes, respectively, following loading with a Ta-emitter solution onto zone-refined outgassed Re-filaments. Analyses of the NIST SRM 987 Sr standard over the course of the measurements yielded values of 0.710264 ± 0.000015 (2 SD; RHUL) and 0.710284 ± 0.000017 (2 SD, OU); consequently, the OU data were adjusted by -0.00002. No Sr concentrations are presented as leaching removes a non-reproducible amount from each sample and no dry weights were recorded following the leaching procedure. Procedure blanks for tooth analysis were ~60 pg Sr and no blank corrections were necessary. Constants used were those from [36].

Eight grams of each soil sample were leached in ~0.25 M acetic acid for one week and shaken regularly. Each leachate was centrifuged, dried down and re-dissolved with ~15 M HNO_3 to remove organics, after which Sr was separated using the same Sr-Spec procedure described above.

In order to minimise modern anthropogenic influence to the highest possible extent, the vegetation samples were collected in forests. Fresh leaves of ground vegetation, mostly, were cleaned using demineralised water, dried overnight at 50°C and then ashed in acid-cleaned silica crucibles (550ºC/12h).

The plant ashes were digested in ultrapure concentrated HNO_3 in teflon beakers on a hot plate at 160°C. Strontium matrix separation was carried out with the Sr-Spec resin on mini-columns (250 µl Resin) using 3 N HNO_3 for loading and washing and H_2O to elute the Sr from the resin. The strontium isotope compositions were measured with TIMS (Sector 54). The TIMS raw data were corrected for a potential rubidium contribution on mass 87 and mass fractionation was correct using an $^{86}Sr/^{88}Sr$ isotope ratio of 0.1194. Finally the $^{87}Sr/^{86}Sr$ isotope ratios were normalised to the widely accepted isotope ratio of 0.71025 for NIST SRM 987. The accuracy of the method used is demonstrated by mean $^{87}Sr/^{86}Sr$ isotope ratios of multiple aliquots of fully processed NIST SRM 987 and the seawater standard IAPSO of 0.710260 (N = 6) and 0.709146 (N = 4) respectively. The expanded analytical uncertainty of the $^{87}Sr/^{86}Sr$ isotope ratios is less than 0.012 % (exemplarily calculated using the IAPSO seawater standard).

The water samples were collected in 30 ml acid-cleaned Teflon tubes and acidified with 100 µl of HNO_3. The strontium isotope ratios of the water samples were obtained using an MC-ICP-MS (VG Axiom) at the Curt-Engelhorn-Center for Archaeometry Mannheim following previously described methods [37, 38]. Raw data were corrected according to the exponential mass fractionation law to $^{88}Sr/^{86}Sr = 8.375209$. The Eimer & Amend standard that was analysed along with the samples yielded a mean of 0.70801 ± 0.00004 (2 SD, n = 45). The inter-laboratory mean is 0.708027 ± 0.000035 (1 SD) [39]. Two aliquots of the same plant sample measured with the TIMS and the MC-ICP-MS provided consistent $^{87}Sr/^{86}Sr$ (difference < 0.00009) values.

References

1. White TD, Folkens PA (2000) Human Osteology. 2. ed., San Diego: Academic Press.

2. Ubelaker DH (1989) Human skeletal remains: Excavation, analysis, interpretation. Washington D.C.: Taraxacum.

3. Meindl RS, Lovejoy CO (1985) Ectocranial suture closure: A revised method for the determination of skeletal age at death based on the lateral-anterior sutures. Am J Phys Anthropol 68: 57-66.

4. Lovejoy CO, Meindl RS, Pryzbeck TR, Mensforth RP (1985) Chronological metamorphosis of the auricular surface of the ilium: A new method for the determination of adult skeletal age at death. Am J Phys Anthropol 68: 15-28.

5. Todd TW (1920) Age changes in the pubic bone I. The male white pubic. Am J Phys Anthropol 3: 285-334.

6. Miles AEW (1963) The dentition in the assessment of individual age in skeletal material. Dent Anthropol 1963: 191-209.

7. Nemeskéri J, Harsanyi L, Ascádi G (1960) Methoden zur Diagnose des Lebensalters von Skelettfunden. Anthropol Anz 24: 70-95.

8. Ferembach D, Schwidetzky I, Stloukal M (1980) Recommendations for age and sex diagnoses of skeletons. J Hum Evol 9: 517-549.

9. Phenice TW (1969) A newly developed visual method of sexing the os pubis. Am J Phys Anthropol 30: 297-301.

10. Novotný V (1972) Geschlechtsmerkmale und Geschlechtsbestimmung auf dem Hüftbein (Os coxae). Konference europskych antrop. Prag.

11. Murail P, Bruzek J, Houët F and Cunha E (2005) DSP: a tool for probabilistic sex diagnosis using worldwide variability in hip-bone measurements. Bulletins et Mémoires de la Société d'Anthropologie de Paris 17: 167-176.

12. Pearson K (1899) Mathematical contributions to the theory of evolution V. On the reconstruction of the stature of prehistoric races. Philos T R Soc Lond A 192: 169-245.

13. Alt KW (1997) Odontologische Verwandtschaftsanalyse. Individuelle Charakteristika der Zähne in ihrer Bedeutung für Anthropologie, Archäologie und Rechtsmedizin. Stuttgart: Fischer.

14. Alt KW, Vach W (1995) Odontologic kinship analysis in skeletal remains: Concepts, methods, and results. Forensic Sci Int 74: 99-113.

15. Aufderheide CA, Rodriguez-Martin C (1998) The Cambridge Encyclopedia of Human Paleopathology. Cambridge: Cambridge University Press.

16. Ortner DJ (2003) Identification of pathological conditions in human skeletal remains. San Diego: Academic Press.

17. Caselitz P (1998) Caries – ancient plague of humankind. In: Alt KW, Rosing FW, Teschler-Nicola M, editors. Dental anthropology. Vienna: Springer. pp. 203-226.

18. Strohm TF, Alt KW (1998) Periodontal disease – etiology, classification and diagnosis. In: Alt KW, Rösing FW, Teschler-Nicola M, editors. Dental anthropology. Vienna: Springer. pp. 227-246.

19. Alt KW, Türp JC, Wächter R (1998) Periapical lesions – clinical and anthropological aspects. In: KW Alt, FW Rösing, M Teschler-Nicola, editors. Dental anthropology. Vienna: Springer. Pp. 247-276.

20. Hengen OP (1971) Cribra orbitalia: Pathogenesis and probable etiology. Homo 22: 57-76.

21. Schultz M, Carli-Thiele P, Schmidt-Schutz TH, Kierdorf U, Kierdorf H, Geegen W-R, Kreutz K (1998) Enamel hypoplasias in archaeological skeletal remains. In: Alt KW, Rösing FW, Teschler-Nicola M, editors. Dental anthropology. Vienna: Springer. pp. 293-311.

22. Lovell NC (1997) Trauma analysis in paleopathology. Yearb Phys Anthropol 40: 139-170.

23. Meyer C, Siebert A, Alt KW (2013) Sturm auf Beda? Bemerkungen zum Nachweis von Spuren der Gewalt am Beispiel der Skelettfunde aus dem spätantik-frühmittelalterlichen Gräberfeld von Bitburg „An der Römermauer". In: Heinrich-Tamáska O, editor. Rauben - Plündern - Morden. Nachweis von Zerstörung und kriegerischer Gewalt im archäologischen Befund. Studien zu Spätantike und Frühmittelalter 5. Hamburg: Dr. Kovač. pp. 67-80.

24. Herrmann B, Grupe G, Hummel S, Piepenbrink H, Schutkowsk H (1990) Prähistorische Anthropologie. Leitfaden der Feld- und Labormethoden. Berlin, Heidelberg: Springer.

25. Haak W, Forster P, Bramanti B, Matsumura S, Brandt G, et al. (2005) Ancient DNA from the first European farmers in 7500-year-old Neolithic sites. Science 310: 1016-1018.

26. Haak W, Brandt G, de Jong HN, Meyer C, Ganslmeier R, et al. (2008) Ancient DNA, strontium isotopes, and osteological analyses shed light on social and kinship organization of the Later Stone Age. Proc Natl Acad Sci 105: 18266-18231.

27. Brandt G, Haak W, Adler CJ, Roth C, Szécsényi-Nagy A, et al. (2013) Ancient DNA reveals key stages in the formation of Central European mitochondrial genetic diversity. Science 342: 257-261.

28. Andrews RM, Kubacka I, Chinnery PF, Lightowlers RN, Turnball DM, et al. (1999) Reanalysis and revision of the Cambridge reference sequence for human mitochondrial DNA. Nature Genet 23: 147.

29. Kloss-Brandstätter A, Pacher D, Schönherr S, Weissensteiner H, Binna R, et al. (2011) HaploGrep: a fast and reliable algorithm for automatic classification of mitochondrial DNA haplogroups. Hum Mutat 32: 25-32.

30. Oven M van, Kayser M (2009) Updated comprehensive phylogenetic tree of global human mitochondrial DNA variation. Hum Mutat 30: E386-E394. http://www.phylotree.org. doi:310.1002/humu.20921.

31. Müldner G, Richards MP (2005) Fast or feast: reconstructing diet in later medieval England by stable isotope analysis. J Archaeol Sci 32: 39-48.

32. Knipper C, Peters D, Meyer C, Maurer A-F, Muhl A, et al. (2013) Dietary reconstruction in Migration Period central Germany: A carbon and nitrogen isotope study. Archaeol Anthropol Sci 5: 17-35.

33. Paul D, Skrzypek G, Fórizs I (2007) Normalization of measured stable isotopic compositions to isotope reference scales – a review. Rapid Commun Mass Sp 21: 3006-3014.

34. Müller W, Fricke H, Halliday AN, McCulloch MT, Wartho J-A (2003) Origin and migration of the Alpine Iceman. Science 302: 862-866.

35. Horn P, Hölzl S, Storzer D (1994) Habitat determination on a dossil stag's mandible from the site of Homo erectus heidelbergensis at Mauer by use of $^{87}Sr/^{86}Sr$. Naturwissenschaften 81: 360-362.

36. Steiger RH, Jäger E (1977) Subcommission on geochronology: Convention on the use of decay constants in geo- and cosmochronology. Earth Planet Sc Lett 36: 359-362.

37. Maurer A-F, Galer SJG, Knipper C, Beierlein L, Nunn EV, et al. (2012) Bioavailable $^{87}Sr/^{86}Sr$ in different environmental samples – Effects of anthropogenic contamination and implications for isoscapes in past migration studies. Sci Total Environ 433: 216-229.

38. Knipper C, Maurer A-F, Peters D, Meyer C, Brauns M, et al. (2012) Mobility in Thuringia or mobile Thuringians: a strontium isotope study from early Medieval central Germany. In: Kaiser E, Burger J, Schier W, editors. Migrations in prehistory and early history. Stable isotopes and population genetics. Berlin: De Gruyter. pp. 293-317.

39. Müller-Sohnius D (2007) $^{87}Sr/^{86}Sr$ for isotope standards of Eimer and Amend (E&A), modern seawater strontium (MSS), and the Standard Reference Material (SRM) 987: development of interlaboratory mean values, procedures of adjusting, and the comparability of results. Geol Bavarica 110: 1-56.
