# Supplementary material for: Lombards on the Move – An Integrative Study of the Migration Period Cemetery at Szólád, Hungary
Source: PLoS One. 2014 Nov 4;9(11):e110793. doi: 10.1371/journal.pone.0110793 (PMC4219681; doi:10.1371/journal.pone.0110793)
Supplement: Text S3 — Results. Osteology. (PDF) [file pone.0110793.s003.pdf]

Test S3: Results

Osteology

Five of the 15 male individuals (33.3 %) from Szólád exhibit traces of traumas caused by violent conflicts or hostilities (Ind. 4, 13, 27, 37, 43). In the Early Middle Ages, grave furnishing with weaponry generally highlights the importance of warfare. Especially the large numbers of weapons in Szólád indicate a highly unstable political situation during the Migration period. The Lombards occupied Pannonia as confederates of Byzantium, and military conflicts with variant confederates and opponents are conveyed for 547, 551-553, 565, and 568 A.D. [1, p. 24-26, p. 150]. The military influence on the society and the political framework combined with the mobile lifestyle possibly contributed to the frequency of fractures among the Szólád burial community. Generally, typical frequencies of skull injuries are 5 – 7 % with a clear predominance of sharp-force traumata [2]. However, exceptions occur, such as the late Antique to Early Medieval cemetery of Bitburg/Germany. There, traces of violence are present at the skulls of 50 % of the burials, but only in males they can be attributed to military activities [3].

Ankylosis of two vertebrae in the central area of the thoracic spine of a male (Ind. 3) was caused by spondylosis degree 4. Two males with fractures on the skulls (Ind. 43) and the humerus (Ind. 45) showed ankyloses of three, resp. four thoracic/lumbar vertebrae which may also have resulted from traumas. Age-related ankyloses are unlikely because the neighboring vertebrae exhibit hardly any alterations. According to the overall diagnostic findings DISH (diffuse idiopathic skeletal hyperostosis) can also be excluded.

References

1. Jarnut J (1982) Geschichte der Langobarden. Stuttgart, Berlin, Köln, Mainz: Kohlhammer.

2. Jakob T (2009) Prevalence and patterns of disease in early Medieval populations. Oxford: Archaeopress.

3. Meyer C, Siebert A, Alt KW (2013) Sturm auf Beda? Bemerkungen zum Nachweis von Spuren der Gewalt am Beispiel der Skelettfunde aus dem spätantik-frühmittelalterlichen Gräberfeld von Bitburg „An der Römermauer“. In: Heinrich-Tamáska O, editor. Rauben - Plündern - Morden Nachweis von Zerstörung und kriegerischer Gewalt im archäologischen Befund. Tagungsbeiträge der Arbeitsgemeinschaft Spätantike und Frühmittelalter 6. Zerstörung und Gewalt im archäologischen Befund (Bremen, 5–6102011). Hamburg: Verlag Dr. Kovač. pp. 67-80.
