# Supplementary material for: Lombards on the Move – An Integrative Study of the Migration Period Cemetery at Szólád, Hungary
Source: PLoS One. 2014 Nov 4;9(11):e110793. doi: 10.1371/journal.pone.0110793 (PMC4219681; doi:10.1371/journal.pone.0110793)
Supplement: Figure S1 — Map of the cemetery of Szólád with indication of anthropological age and sex determinations and results of aDNA and strontium isotope analysis. (PDF) [file pone.0110793.s005.pdf]

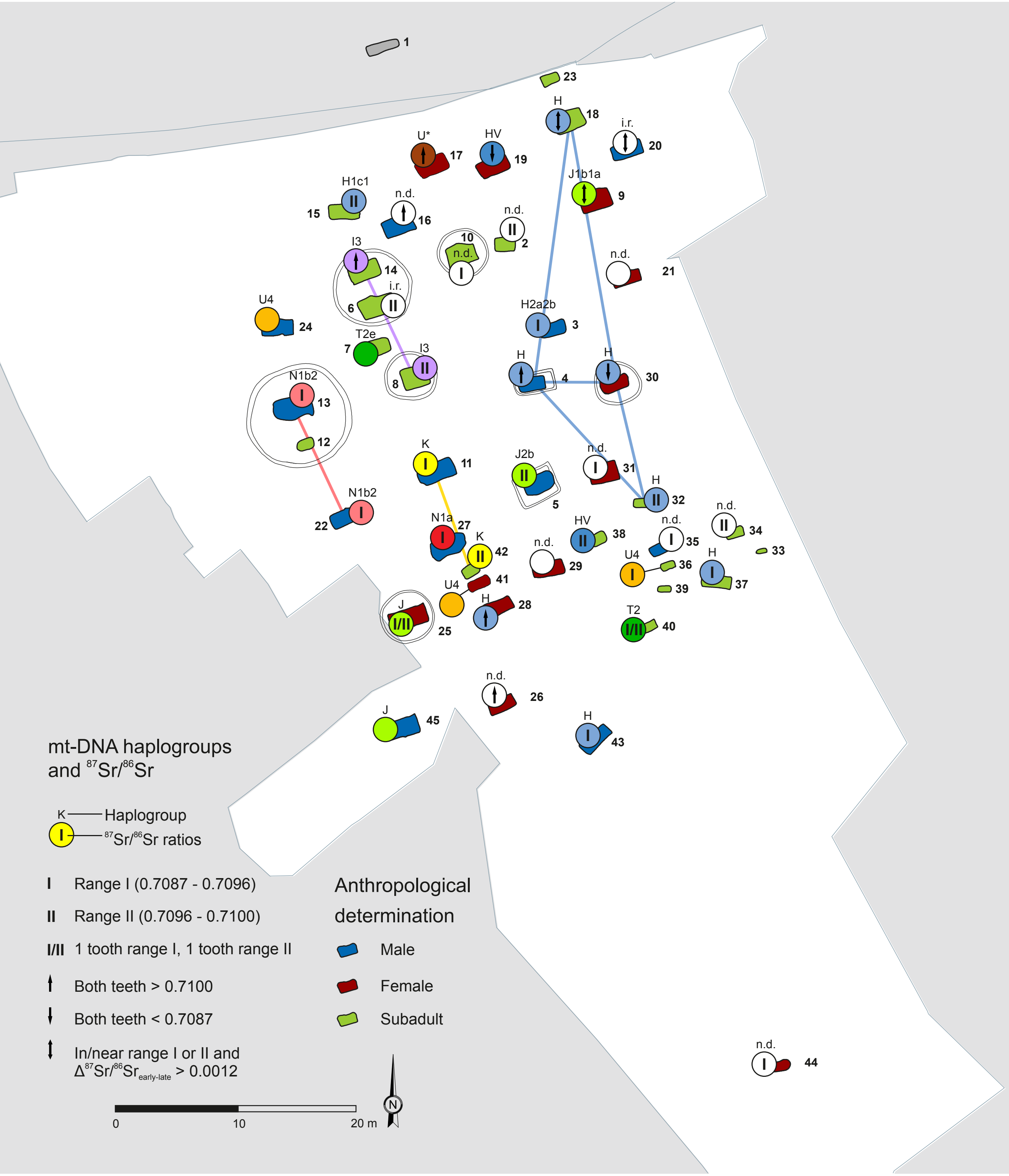

Figure S1: Map of the cemetery of Szólád with indication of anthropological age and sex determinations and results of aDNA and strontium isotope analyses. The colours of the circles indicate mt-DNA haplogroups that are named above the circles. The symbols in the circles indicate the results of Sr isotope analyses. Connected individuals have identical mt-DNA haplotypes.
